# Estimating search engine index size variability: a 9-year longitudinal study

**DOI:** 10.1007/s11192-016-1863-z

**Published:** 2016-02-09

**Authors:** Antal van den Bosch, Toine Bogers, Maurice de Kunder

**Affiliations:** Centre for Language Studies, Radboud University, PO Box 9103, 6500 HD Nijmegen, The Netherlands; Department of Communication, Aalborg University Copenhagen, A.C. Meyers Vænge 15, 2450 Copenhagen, Denmark; De Kunder Internet Media, Toernooiveld 100, 6525 EC Nijmegen, The Netherlands

**Keywords:** Search engine index, Webometrics, Longitudinal study

## Abstract

One of the determining factors of the quality of Web search engines is the size of their index. In addition to its influence on search result quality, the size of the indexed Web can also tell us something about which parts of the WWW are directly accessible to the everyday user. We propose a novel method of estimating the size of a Web search engine’s index by extrapolating from document frequencies of words observed in a large static corpus of Web pages. In addition, we provide a unique longitudinal perspective on the size of Google and Bing’s indices over a nine-year period, from March 2006 until January 2015. We find that index size estimates of these two search engines tend to vary dramatically over time, with Google generally possessing a larger index than Bing. This result raises doubts about the reliability of previous one-off estimates of the size of the indexed Web. We find that much, if not all of this variability can be explained by changes in the indexing and ranking infrastructure of Google and Bing. This casts further doubt on whether Web search engines can be used reliably for cross-sectional webometric studies.

## Introduction

Webometrics (or cybermetrics) is commonly defined as the study of the content, structure, and technologies of the World Wide Web (WWW) using primarily quantitative methods. Since its original conception in 1997 by Almind & Ingwersen, researchers in the field have studied aspects such as the link structure of the WWW, credibility of Web pages, Web citation analysis, the demographics of its users, and search engines (Thelwall 2009). The size of the WWW, another popular object of study, has typically been hard to estimate, because only a subset of all Web pages is accessible through search engines or by using Web crawling software. Studies that attempt to estimate the size of the WWW tend to focus on the surface Web—the part indexed by Web search engines—and often only at a specific point in time.

In the early days of search engines, having the biggest index size provided search engines with a competitive advantage, but a changing focus on other aspects of search result quality, such as recency and personalization, has diminished the importance of index size in recent years. Nevertheless, the size of a search engine’s index is important for the quality of Web search engines, as argued by Lewandowski and Höchstötter (2008). In addition, knowledge of the size of the indexed Web is important for webometrics in general, as it gives us a ceiling estimate of the size of the WWW that is accessible by the average Internet user.

The importance of index sizes in the early days of Web search resulted in several estimation methods, most of which used the overlap between different Web search engines to estimate the size of the indexed Web as a whole. Bharat and Broder (1998) used an overlap-based method to estimate the size of the WWW at around 200 million pages. Lawrence and Giles (1998, 1999) produced higher estimates of 320 and 800 million pages in 1998 and 1999 using a similar method. Six years later, Gulli and Signorini (2005) updated these estimates to 11.5 billion pages. The last decade has seen little work on index size estimation, but a general problem with all of the related work so far is that all the analyses have been cross-sectional. There has been no analysis of index size on a longer time scale that sheds light on the robustness of any of estimation methods used. The handful of studies that have taken a longer-term perspective have typically focused on Web page persistence (Koehler 2004) or academic link structure (Payne and Thelwall 2008), but never on search engine index size.

In this paper we present a novel method of estimating the size of a Web search engine’s index by extrapolating from document frequencies of words observed in a large static corpus of Web pages. In addition, we provide a unique longitudinal perspective on our estimation method by applying it to estimate the size of Google and Bing’s^1^ indices over a period of close to nine years, from March 2006 until January 2015. More specifically, we attempt to answer the following research questions:

**RQ 1**: How can the index size of a Web search engine be estimated reliably using document frequency extrapolation on an external corpus?
**RQ 2**: How has the index size of Google and Bing developed over the past 9 years?

We find that index size estimates of these two search engines tend to vary wildly over time, with Google generally possessing a larger index than Bing. This considerable variability has been noted in earlier work (e.g., Rousseau 1999; Payne and Thelwall 2008), which raises doubts about the reliability of previous one-off estimates of the size of the indexed Web. In our analysis, we find that much of this variability can be explained by changes in the indexing and ranking infrastructure of Google and Bing. This casts further doubt on whether the hit counts from Web search engines can be used reliably for cross-sectional Webometric studies, confirming similar sentiments expressed by, for instance, Payne and Thelwall (2008) and Thelwall and Sud (2012).

The remainder of this paper is organized as follows. The next section contains a review of related work in webometrics and on estimating the size of the indexed WWW. We then explain our estimation method in more detail, followed by the results of our estimation method and an analysis of the intrinsic and extrinsic variability we uncover in our estimation results. We then discuss our findings using a list of potential measurement biases, draw our conclusions, and formulate points for future research.

## Related work

Since its inception, researchers have studied many different aspects of the Web. This section provides a brief overview of some of the key studies on measuring different properties of Web search engines and the WWW, in particular work on estimating their size.

### Measuring the web

Over the past two decades many aspects of the WWW have been studied, such as the link structure of the Web that emerges from the hyperlinks connecting individual Web pages. Broder et al. (2000) were among the first to map the link structure of the WWW. They showed that the Web graph can be visualized as a bow-tie structure with 90 % of all pages being a part of the largest strongly connected component, a hypothesis which was confirmed in 2008 by Hirate et al. (2008).

Payne and Thelwall (2008) performed a longitudinal analysis of hyperlinks on academic Web sites in the UK, Australia, and New Zealand over a six-year period. They found that the inlink and outlink counts were relatively stable over time, albeit with large fluctuations at the individual university level. As a result, they concluded that such variability could create problems for the replicability and comparability of webometrics research. Other related work on analyzing the link structure of the Web includes Kleinberg et al. (1999) and Björneborn (2004).

*Search engines* Web search engines are an essential part of navigating the WWW and as a result are have received much attention. Many different aspects of Web search have been investigated, such as ranking algorithms, evaluation, user behavior, and ethical and cultural perspectives. Bar-Ilan (2004) and Zimmer (2010) provide clear, multi-disciplinary overviews of the most important work on these aspects. From a webometric perspective the hit counts, search engine rankings, and the persistence of the indexed URLs are highly relevant for the validity and reliability of webometric research using Web search engines.

*Hit counts* Rousseau (1999) was among the first to investigate the stability of search engine results by tracking the hit counts—the number of results indicated for a query—for three single-word search terms in Altavista and NorthernLight over a 12-week period in 1998. His choice of the “neutral” words *saxophone*, *trumpet*, and *pope* is likely to have introduced several biases. Nevertheless, Altavista exhibited great variability over a longer time period, even with only three anecdotal query words. Rousseau attributed this to changes in Altavista’s infrastructure with the launch of a new version in 1998.

Thelwall (2008) also performed a cross-sectional quantitative comparison of the hit counts and search engine results of Google, Yahoo!, and Live Search (Thelwall 2008). He extracted 1587 single-word queries from English-language blogs “based purely on word frequency criteria” (Thelwall 2008, p. 1704) and found strong correlations between the hit count estimates of all three search engines, and recommended using Google for obtaining accurate hit count estimates, albeit with large differences in orders of magnitude between the actual counts. In contrast, the number of different URLs, sites, and domains returned by the three search engines showed a considerable amount of variation and inconsistency. Thelwall’s final recommendation is to use Google for obtaining accurate hit count estimates, something we have done in our approach.

Uyar (2009) extended Thelwall’s work by including multi-word queries. He found that the number of words in the query significantly affects the accuracy of hit counts, with single-word queries providing nearly double the hit count accuracy as compared to multi-word queries.

Finally, Thelwall and Sud (2012) investigated the usefulness of version 2.0 of the Bing Search API for performing webometric research. They examined, among other things, the hit count estimates, and found that these can vary by up to 50 % and, as a result, caution against using them in webometric research.

*Search engine rankings* Bar-Ilan et al. (2006) compared the rankings of three different Web search engines over a three-week period. They observed that the overlap in result lists for textual queries was much higher than for image queries, where the result lists of the different search engines showed almost no overlap. Spink et al. (2006) investigated the overlap between three major Web search engines based on the first results pages and found that 85 % of all returned top-10 results are unique to that search engine.

Vaughan and Thelwall (2004) examined the national biases of Google, Altavista and AllTheWeb towards websites from four different countries: the United States, China, Taiwan, and Singapore. They found that all search engines showed significant differences in coverage of commercial Web sites, with US websites having a strong advantage over the other three. They argued that this was mostly due to the increased visibility of these sites in terms of incoming links as opposed to a deliberate bias on the part of the search engines.

*Web page persistence* The issue of Web page persistence in search engine indices—how long does a Web page remain indexed and available—was first examined by Bar-Ilan (1999) for a single case-study query during a five-month period in 1998. She found that for some search engines up to 60 % of the results had disappeared from the index at the end of the period. She hypothesized that the distributed nature of search engines may cause different results to be served up from different index shards at different points in time. A similar study by Bar-Ilan (2004) presented the results of a five-year longitudinal study on the persistence and evolution of Web pages on the topic of “informetrics” Bar-Ilan (2004). They found that as much as 40 % of all original URLs has disappeared after 5 years.

Koehler (2004) reported on the results of a six-year longitudinal study on Web page persistence. He also provided an overview of different longitudinal studies on the topic and concluded, based on the relatively small number of studies that exist, that Web pages are not a particularly persistent medium, although there are meaningful differences between navigation and content pages.

### Index size estimation

In the last two decades, various attempts have been directed at estimating the size of the indexed Web. Some approaches focus on estimating the index size of a single search engine directly, while a majority focuses on estimating the overlap to indirectly estimate the size of the total indexed Web.

Highly influential work on estimating index size was done in 1997 by Bharat and Broder (1998), who attempted to calculate the relative sizes and overlaps of search engine indices in two steps. By randomly selecting pages from the index of a particular engine and checking whether they occur in the index of another engine and vice versa, the size ratio can be computed from the overlap estimates (Bharat and Broder 1998). Collecting these overlap counts requires two steps: randomly selecting a page from one engine, and checking whether the page was indexed by another engine. In the sampling phase, Bharat and Broder had to use the search engines themselves to generate the random samples because of practical reasons. They collected a 400,000 word lexicon by crawling the 300,000 pages the Yahoo! Directory contained in 1997, and generated 35,000 random conjunctive and disjunctive queries from 6 to 8 randomly selected words in this lexicon. These queries were sent to 4 search engines—HotBot, AltaVista, Excite, Infoseek—after which one of the top-100 result pages was randomly selected. Selecting a random page from the entire set of results would have prevented the ranking bias that this method introduces, but “search engines usually do not return more than a few hundred matches and in fact may not even compute the remaining matches” (Bharat and Broder 1998, p. 382).

In the next phase, they checked for the presence of each of these randomly selected pages in the other engines’ indices. By taking the top *k* most discriminant terms from each randomly selected page, they constructed so-called *strong* queries, meant to uniquely identify the randomly selected page in the other engines’ indices. If a result URL of that strong query matched the URL of the randomly selected page after normalization, it was counted as present in the other engine’s index. This imperfect process introduced an additional, checking bias to their approach (in Sect. 5.1 we further discuss the types of bias encountered by our approach). Using the reported index sizes from the search engines they estimated the size of the indexed WWW in 1997 to be around 200 million pages (Bharat and Broder 1998).

Gulli and Signorini (2005) extended the work of Bharat and Broder (1998) by increasing the number of submitted queries by an order of magnitude, and using 75 different languages. They calculated the overlap between Google, Yahoo!, MSN Live, and Ask.com, and updated the previous estimates to 11.5 billion pages in January 2005. Most approaches that use the work of Bharat and Broder as a starting point focus on improving the sampling of random Web pages, which can be problematic because not every page has the same probability of being sampled using Bharat and Broder’s approach. Several researchers have proposed methods of near-uniform sampling that attempt to compensate for this ranking bias, such as Henzinger et al. (2000), Anagnostopoulos et al. (2006), and Bar-Yossef and Gurevich (2006, 2011).

Lawrence and Giles (1998) estimated the indexed overlap of six different search engines. They captured the queries issued by the employees of their own research institute and issued them to all six engines. The overlap among search engines was calculated on the aggregated result sets, after which they used publicly available size figures from the search engines to estimate the size of the indexed Web to be 320 million pages in 1998. Lawrence and Giles updated their previous estimates to 800 million Web pages in July 1999 (Lawrence and Giles 1999). Dobra and Fienberg (2004) used statistical population estimation methods to improve upon the original 1998 estimate of Lawrence and Giles. They estimated that Lawrence and Giles were off by a factor two and that the Web contained around 788 million Web pages in 1998. Khelghati et al. (2012) compared several of the aforementioned estimation methods as well as some proposed modifications to these methods. They found that a modified version of the approach proposed by Bar-Yossef and Gurevich (2011) provided the best performance.

## Estimating the size of a search engine through extrapolation

The method we propose in this contribution, based on and extending an earlier version of this work (Van den Bosch et al. 2015), does not use the overlap between search engines on the basis of a near-uniform sampling of web documents, but rather uses a sampled web page collection as the basis for extrapolating an estimate of the number of documents in a search engine’s index.

In the following subsections we describe in detail how we estimated word frequencies from a fully known training corpus in order to estimate the unknown size of a test corpus, given the frequency of a word in the test corpus. We introduce DMOZ, the selected training corpus. We explain how we took the arithmetic mean over 28 selected pivot words, and we describe how we started the longitudinal (and still running) experiment in March 2006.

### Estimating word frequencies for corpus size extrapolation

On the basis of a textual corpus that is fully available, both the number of documents and the term and document frequencies of individual terms can be counted. In the context of Web search engines, however, we only have reported hit counts (or document counts), and we are usually not informed about the total number of indexed documents. Since it is the latter we are interested in, we want to estimate the number of documents indexed by a search engine indirectly from the reported document counts.

We can base such estimates on a training corpus for which we have full information on document frequencies of words and on the total number of documents. From the training corpus we can extrapolate a size estimation of any other corpus for which document counts are given. Suppose that, for example, we collect a training corpus *T* of 500,000 web pages, i.e. $|T| = 500,000$. For all words *w* occurring on these pages we can count the number of documents they occur in, or their document count, $d_T(w)$. A frequent word such as *are* may occur in 250,000 documents, i.e. it occurs in about one out of every two documents; $d_T(are) = 250,000$. Now if the same word *are* is reported to occur in 1 million documents in another corpus *C*, i.e. its document count $d_C(are) = 1,000,000$, we can estimate by extrapolation that this corpus will contain about $|C| = \frac{d_C(are) \times |T|}{d_T(are)}$, i.e. 2 million documents.

There are two crucial requirements that would make this extrapolation sound. First, the training corpus would need to be representative of the corpus we want to estimate the size of. Second, the selection of words^2^ that we use as the basis for extrapolation will need to be such that the extrapolations based on their frequencies are statistically sound. We should not base our estimates on a small selection of words, or even a single word, as frequencies of both high-frequency and low-frequency words may differ significantly among corpora. Following the most basic statistical guidelines, it would be better to repeat this estimation for several words, and average over the extrapolations. The number of words *n* should be high enough such that the arithmetic means of different selections of *n* words are normally distributed around an average, according to the Central Limiting Hypothesis (Rice 2006).

A random selection of word types is likely to produce a selection with relatively low frequencies, as Zipf’s second law predicts (Zipf 1935). A well-known issue in corpus linguistics is that when any two corpora are different in genre or domain, very large differences are likely to occur in the two corpora’s word frequencies and document frequencies, especially in the lower frequency bands (or the long tails) of the term distributions. It is not uncommon that half of the word types in a corpus occur only once; many of these terms will not occur in another disjoint corpus, even if it represents the same genre. This implies that extrapolations should not be based on a random selection of terms, many of which will have a low frequency of occurrence. The selection of words should sample several high-frequency words but preferably also several other words with frequencies spread across the other frequency bands.

It should be noted that Zipf’s law concerns word frequencies, not document frequencies. Words with a higher frequency tend to recur more than once in single documents. The higher the frequency of a word, the more its document frequency will be lower than its word frequency. A ceiling effect thus occurs with the most frequent words if the corpus contains documents of sufficient size: they tend to occur in nearly all documents, making their document frequencies within the same order of magnitude as the number of documents in the corpus, while at the same time their word token frequencies still differ to the degree predicted by Zipf’s law (Zipf 1935). This fact is not problematic for our estimation goal, but it should be noted that this hinges on the assumption that the training corpus and the new corpus of which the frequencies are unknown, contain documents of about the same average size.

### Selecting a representative corpus: DMOZ

As our purpose is to estimate the size of a Web search engine’s index, we must make sure that our training corpus is representative of the web, containing documents with a representative average size. This is quite an ambitious goal. We chose to generate a randomly filtered selection of 531,624 web pages from the DMOZ^3^ web directory. We made this selection in the spring of 2006. To arrive at this selection, first a random selection was made of 761,817 DMOZ URLs, which were crawled. Besides non-existing pages, we also filtered out pages with frames, server redirects beyond two levels, and client redirects. In total, the resulting DMOZ selection of 531,624 documents contains 254,094,395 word tokens (4,395,017 unique word types); the average DMOZ document contains 478 words.

We then selected a sequence of *pivot* words by their frequency rank, starting with the most frequent word in the DMOZ data, and selecting an exponential series where we increase the selection rank number with a low exponent, viz. 1.6. We ended up with a selection of the following 28 pivot words, the first nine being high-frequency function words and auxiliary verbs: *and, of, to, for, on, are, was, can, do, people, very, show, photo, headlines, william, basketball, spread, nfl, preliminary, definite, psychologists, vielfalt, illini, chèque, accordée, reticular, rectificació*. The DMOZ directory is multilingual, but English dominates. It is not surprising that the tail of this list contains words from different languages.

Our estimation method then consists of retrieving document counts for all 28 pivot words from the search engine we wish to estimate the number of documents for, obtaining an extrapolated estimate for each word, and taking an arithmetic mean over the 28 estimations. If a word is not reported to occur in any document (which hardly happens with Google or Bing), it is not included in the average. In preliminary experiments, we tested different selections of 28 words using different starting words but the same exponential rank factor of 1.6, and found closely matching averages of the computed extrapolations.

To stress-test the assumption that the DMOZ document frequencies of our 28 pivot words yield sensible estimates of corpus size, we estimated the size of a range of corpora: the New York Times part of the English Gigaword corpus^4^ (newspaper articles published between 1993 and 2001), the Reuters RCV1 corpus^5^ (newswire articles), the English Wikipedia^6^ (encyclopedic articles, excluding pages that redirect or disambiguate), and a held-out sample of random DMOZ pages (not overlapping with the training set, but drawn from the same source). If our assumptions are correct, the size of the latter test corpus should be fairly accurate. Table 1 provides an overview of the estimations on these widely different corpora. The size of the New York Times corpus is overestimated by a large margin of 126 %. The size of the Wikipedia corpus is only mildly overestimated by 3.6 %. The sizes of the Reuters and DMOZ corpora are underestimated. The size of the DMOZ sample is indeed relatively accurately estimated, with a small underestimation of 1.3 %.

**Table 1:** Real versus estimated numbers (with standard deviations) of documents on four textual corpora, based on the DMOZ training corpus statistics: two news resources (top two) and two collections of web pages (bottom two)

Corpus	Words per document					
	Mean	Median	Number of # documents	Estimate	SD	Difference (%)
New York times	837	794	1,234,426	2,789,696	1,821,823	+126
Reuters RCV1	295	229	453,844	422,271	409,648	−7.0
Wikipedia	447	210	2,112,923	2,189,790	1,385,105	+3.6
DMOZ test sample	477	309	19,966	19,699	5,839	−1.3

### Taking the arithmetic mean

**Fig. 1:**
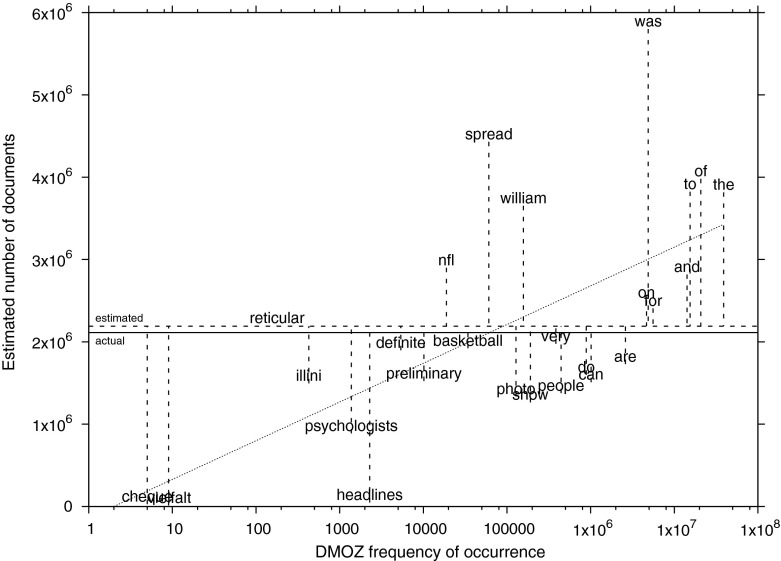
Labeled scatter plot of per-word DMOZ frequencies of occurrence and estimates of the Wikipedia test corpus. The x axis is logarithmic. The *solid horizontal line* represents the actual number of documents in the Wikipedia test corpus (2,112,923); the *dashed horizontal line* is the averaged estimate of 2,189,790. The *dotted slanted line* represents the log-linear regression function $x = (204,224 \times ln(x)) - 141,623$

The standard deviations of the averages listed in Table 1, computed over the 28 pivot words, indicate that the per-word estimates are dispersed over quite a large range. Figure 1 illustrates this for the case of the Wikipedia corpus (the third data line of Table 1). There is a tendency for the pivot words in the highest frequency range (*the, of, to*, and especially *was*) to cause overestimations, but this is offset against relatively accurate estimates from pivot words with a mid-range frequency such as *very*, *basketball*, and *definite*, and underestimations from low-frequency words such as *vielfalt* and *chèque*. The DMOZ frequency of occurrence and the estimated number of documents in Wikipedia are only weakly correlated, with a Pearson’s $R=0.48,$ but there is an observable trend in low frequencies causing underestimations, and high frequencies causing overestimations. The log-linear regression function with the smallest residual sum of squares is the function $x = (204,224 \times ln(x)) - 141,623,$ visualized as the slanted dotted line in Fig. 1. Arguably, selecting exponentially-spaced pivot words across the whole frequency spectrum leads to a large standard deviation, but a reasonably accurate mean estimate on collections of web pages.

### Setting up the longitudinal experiment

After having designed this experiment in March 2006, we started to run it on a daily basis on March 13, 2006, and have done so ever since.^7^ Each day we send the 28 DMOZ words as queries to two search engines: Bing and Google.^8^ We retrieve the reported number of indexed pages on which each word occurs (i.e., the hit counts) as it is returned by the web interface of both search engines, not their APIs. These hit counts were extracted from the first page of results using regular expressions. This hit count is typically rounded: it retains three or four significant numbers, the rest being padded by zeroes. For each word we use the reported document count to extrapolate an estimate of the search engine’s size, and average over the extrapolations of all words. The web interfaces to the search engines have gone through some changes, and the time required to adapt to these changes sometimes caused lags of a number of days in our measurements. For Google 3027 data points were logged, which is 93.6 % of the 3235 days between March 13, 2006 and January 20, 2015. For Bing, this percentage is 92.8 % (3002 data points).

## Results

**Fig. 2:**
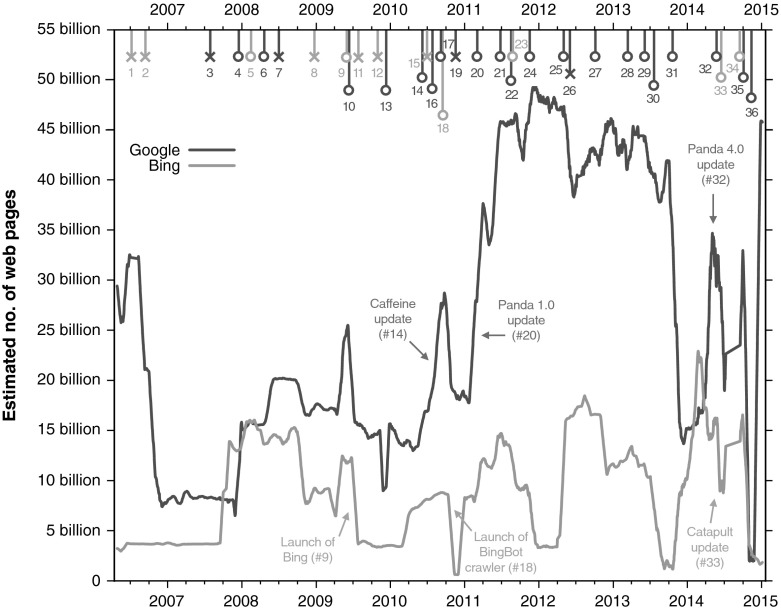
Estimated size of the Google and Bing indices from March 2006 to January 2015. The *lines* connect the unweighted running daily averages of 31 days. The *colored*, *numbered markers* at the top represent reported changes in Google and Bing’s infrastructure. The *colors* of the *markers* correspond to the color of the search engine curve they related to; for example, *red markers* signal changes in Google’s infrastructure (the *red curve*). Events that line up with a spike are marked with an *opened circle*, other events are marked with an *times*

Figure 2 displays the estimated sizes of the Google and Bing indices between March 2006 and January 2015. For visualization purposes and to avoid clutter, the numbers are unweighted running averages of 31 days, taking 15 days before and after each focus day as a window. The final point in our measurements is January 20, 2015; hence the last point in this graph is January 5, 2015. Rather than a linear, monotonic development we observe a rather varying landscape, with Google usually yielding the larger estimates. The largest peak in the Google index estimates is about 49.4 billion documents, measured in mid-December 2011. Occasionally, estimates are as low as under 2 billion pages (e.g. 1.96 billion pages in the Google index on November 24, 2014), but such troughs in the graph are usually short-lived, and followed by a return to high numbers (e.g., to 45.7 billion pages in the Google index on January 5, 2015).

### Intrinsic variability

**Fig. 3:**
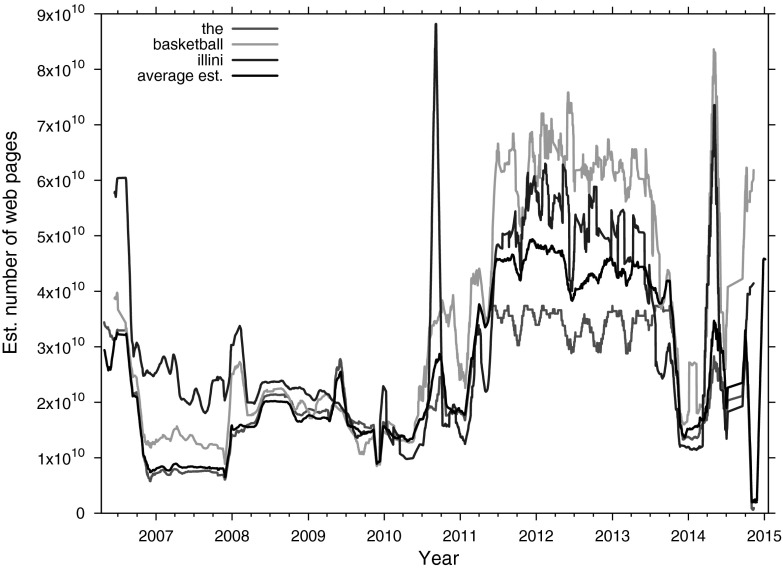
Estimated size of the Google index from March 2006 to January 2015 for three pivot words, *the*, *basketball*, and *illini*, and the average estimate over all 28 words (*black line*). The *lines* connect the unweighted running daily averages of 31 days

The average estimate computed on the basis of the individual estimates of the 28 pivot words, displayed in Fig. 2, has a relatively high standard deviation, as the test results in Table 1 already indicated. To ascertain the source of this variability it is important to check whether individual differences between pivot-word-based estimates also vary over time; perhaps the large fluctuations are caused by individual variations of per-word estimates over time. Figure 3 visualizes the estimate of the size of the Google index for a number of words: the pivot word with the highest frequency, *the*; a word from the mid-frequency range, *basketball*, and a low-frequency word, *illini*. The most frequent word *the* occurs in 359,419 of the 531,624 DMOZ documents; *basketball* occurs in 5183 documents, and *illini* occurs only in 86 documents. The black line in Fig. 3 represents the average over all 28 pivot words for the Google index, already displayed in Fig. 2. How do the averages of the three example pivot words relate to this average? We observe the following:

- The graphs for all three words show similar overall trends, and small individual variations;
- The graph of the pivot word *the* follows the overall estimate quite closely, except for the period mid-2011 to the end of 2013. In this period, the estimate for *the* is roughly 20 % under the overall average;
- The graph of *basketball* mostly follows the average. In the same period where *the* produces sub-average estimates, *basketball* produces larger numbers, exceeding the average by 10 to 20 billion pages;
- The graph of *illini* is generally close to the average after the beginning of 2008, but exhibits two marked peaks, the second of which aligns with a marked peak of the estimate of the word *basketball*;
- The overestimations and underestimations observed earlier with the Wikipedia test corpus with high and low-frequency words, respectively, do not hold with these three example words.

Overall, this analysis of the intrinsic variability of the components of the average estimate indicate that the individual words follow an overall trend; Google is reporting document counts that go up and down over time for all words simultaneously.

### Extrinsic variability

The variability observed in Fig. 2 is not surprising given the fact that the indexing and ranking architectures of Web search engines are updated and upgraded frequently. According to Matt Cutts,^9^ Google makes “roughly 500 changes to our search algorithm in a typical year”, and this is likely the same for Bing. While most of these updates are not publicized, some of the major changes that Google and Bing make to their architectures are announced on their official blogs. To examine which spikes and steps in Fig. 2 can be attributed to publicly announced architecture changes, we went through all blog posts on the Google Webmaster Central Blog,^10^ the Google Official Blog,^11^ the Bing Blog,^12^ and Search Engine Watch^13^ for reported changes to their infrastructure. This resulted in a total of 36 announcements related to changes in the indexing or ranking architecture of Google and Bing.^14^ The colored, numbered markers at the top of Fig. 2 show how these reported changes are distributed over time.

For Google 20 out of the 24 reported changes appear to correspond to sudden spikes and steps in the estimated index size, and for Bing 6 out of 12 reported changes match up with estimation spikes and steps. This strongly supports the idea that much of the variability can be attributed to such changes. Examples include the launch of Bing on May 28, 2009 (event #9), the launch of Google’s search index Caffeine on June 8, 2010 (event #14), the launch of the BingBot crawler (event #18), and the launches of Google Panda updates, and Bing’s Catapult update (events #20, #32, and #33).

Not all sudden spikes and steps can be explained by reported events. For example, the spike in Bing’s index size in October 2014 does not match up with any publicly announced changes in their architecture, although it is a likely explanation for such a significant change. In addition, some changes to search engine architectures are rolled out gradually and would therefore not translate to spikes in the estimated size. However, much of the variation in hit counts, and therefore estimated index size, appears to be caused by changes in the search engine architecture–something already suggested by Rousseau in his 1999 study.

## Discussion and conclusions

In this article we presented a method for estimating the size of a Web search engine’s index. Based on the hit counts reported by two search engines, Google and Bing, for a set of 28 words the size of the index of each engine is extrapolated (RQ 1). We repeated this procedure and performed it once per day, starting in March 2006; it has not stopped functioning so far. Answering our second research question RQ2, “How has the index size of Google and Bing developed over the past 9 years?”, the results do not show a steady, monotonic growth, but rather a highly variable estimated index size. The larger estimated index of the two, the one from Google, attains high peaks of close to 50 billion web pages, but occasionally drops to small indices of 2 billion pages as well. Are we measuring the extrinsic variability of the indices, or an intrinsic variability of our method? Our method is fixed: the same 28 words are sent to both search engines on every day. The frequencies of our test words are unlikely to change dramatically in a corpus as big as a crawl of the indexed Web; especially the document counts for our high-frequent words in our list should be in the same order of magnitude as the total number of documents in the index. Indeed, as Fig. 3 supports, we see that the individual estimates for pivot words follow the same overall trends; if Google reports higher or lower document counts over time, it does so for all words.

We argue that this variability we measure is largely—if not entirely—attributable to the variability of the index of Google and Bing. In other words, what we are measuring is the genuine extrinsic variability of the indices, caused by changes (e.g., updates, upgrades, overhauls) of the indices. In Fig. 2 we highlighted several publicly announced changes to both search engines’ indices, many of which co-occur with drastic changes in index size as estimated by our method (20 out of the 24 reported changes in the Google index, and 6 out of 12 changes in Bing’s index).

This variability, noted earlier also by Rousseau (1999), Bar-Ilan (1999), and Payne and Thelwall (2008), should be a cause for concern for any non-longitudinal study that adopts reported hit counts. It has been pointed out that “Googleology is bad science” (Kilgarriff 2007), meaning that commercial search engines exhibit variations in their functioning that do not naturally link to the corpus they claim to index. Indeed, it is highly unlikely that the real indexable Web suddenly increased from 20 to 30 billion pages in a matter of weeks in October 2014; yet, both the Bing and Google indices report a peak in that period.

Our estimates do not show a monotonic growth of Web search engines’ indices, which was one of the hypothesized outcomes at the onset of this study in 2006. The results could be taken to indicate that the indexed Web is not growing steadily the way it did in the late 1990s. They may even be taken to indicate the indexed Web is not growing at all. Part of this may relate to the growth of the unindexed Deep Web, and a move of certain content from the indexed to the Deep Web.

### Limitations

While our approach provides a unique perspective on the variability of hit counts due to its longitudinal nature, it also has certain limitations and inherent biases that should be mentioned. It is important to note, for instance, that the observed instability of hit counts over a longer period of time does not automatically carry over to other types of webometric analyses. Measuring other properties of search engines for use in webometric research, such as result rankings or link structure, do not suffer from the same problems we highlighted.

Moreover, we extracted our hit counts directly from the search engine results pages. Bing, however, also offers a public API^15^ for access to search results and hit counts. While it is certainly possible that the hit counts provided by this API differ from the ones provided through the public Web interface, we do believe this to be highly unlikely. Additional evidence for the hit count variability also being present in the Bing API has been provided by Thelwall and Sud (2012), who reported API hit counts could vary by up to 50 %. In short, our recommendation is to use the hit counts reported by search engines for webometric research with great caution. Related work seems to suggests that other forms of webometric analyses fare better with the Bing API.

Any approach to index size estimation suffers from different types of biases. For the sake of completeness, we list here a number of possible biases from the literature and how they apply to our own approach:

*Query bias*: According to Bharat and Broder (1998), large, content-rich documents have a better chance of matching a query. Since our method of absolute size estimation relies on the hit counts returned by the search engines, it does not suffer from this bias, as the result pages themselves are not used.
*Estimation bias*: Our approach relies on search engines accurately reporting the genuine document frequencies of all query terms. However, modern search engines tend to not report the actual frequency, but instead estimate these counts, for several reasons. One such reason is their use of federated indices: a search engine’s index is too large to be stored on one single server, so the index is typically divided over many different servers. Update lag or heavy load of some servers might prevent a search engine from being able to report accurate, up-to-date term counts. Another reason for inaccurate counts is that modern search engines tend to use document-at-a-time (DAAT) processing instead of term-at-a-time (TAAT) processing (Turtle and Flood 1995). In TAAT processing the postings list is traversed for each query term in its entirety, disregarding relevant documents with each new trip down the postings list. In contrast, DAAT processing the postings list is traversed one document at a time for all query terms in parallel. As soon as a fixed number of relevant documents—say 1000—are found, the traversal is stopped and the resulting relevant documents are returned to the user. The postings list is statically ranked before traversal (using measures such as PageRank) to ensure high-quality relevant documents. Since DAAT ensures that, usually, the entire postings list does not have to be traversed, the term frequency counts tend to be incomplete. Therefore, the term frequencies are typically estimated from the section of the postings list that was traversed.
*Malicious bias*: According to Bharat and Broder (1998, p. 384), a search engine might rarely or never serve pages that other engines have, thus completely sabotaging our approach. This unlikely scenario is not likely to influence our approach negatively. However, if search engines were to maliciously inflate the query term counts, this would seriously influence our method of estimating the absolute index sizes.
*Domain bias*: By using text corpora from a different domain to estimate the absolute index sizes, a domain bias can be introduced. Because of different terminology, term statistics collected from a corpus of newswire, for instance, would not be applicable for estimating term statistics in a corpus of plays by William Shakespeare or corpus of Web pages. We used a corpus of Web pages based on DMOZ, which should reduce the domain bias considerably. However, in general the pages that are added to DMOZ are of high quality, and are likely to have a higher-than-average PageRank. Potentially their high rank is related to a richer type of textual content, which might produce overestimations. We have not compared our random DMOZ corpus against a near-uniformly sampled web corpus.
*Cut-off bias*: Some search engines typically do not index all of the content of all web pages they crawl. Since representative information is often at the top of a page, partial indexing does not have adverse effect on search engine performance. However, this cut-off bias could affect our term estimation approach, since our training corpus contains the full texts for each document. Estimating term statistics from, say, the top 5 KB of a document can have a different effect than estimating the statistics from the entire document. Unfortunately, it is impractical to figure out what cut-off point the investigated search engines use so as to replicate this effect on our training corpus.
*Quality bias*: DMOZ represents a selection of exemplary, manually selected web pages, while it is obvious that the web at large is not of the same average quality. Herein lies a bias of our approach. Some aspects of the less representative parts of the web have been identified in other work. According to Fetterly et al. (2005), around 33 % of all Web pages are duplicates of one another. In addition, in the past about 8 % of the WWW was made up of spam pages (Fetterly et al. 2005). If this is all still the case, this would imply that over 40 % of the Web does not show the quality nor the variation present in the DMOZ training corpus.
*Language bias*: Our selection of words from DMOZ are evenly spread over the frequency continuum and show that DMOZ is biased towards the English language, perhaps more than the World Wide Web at large. A bias towards English may imply an underestimation of the number of pages in other languages, such as Mandarin or Spanish.
*Statistical sampling error bias*: As mentioned by Bharat and Broder, when estimating a measurement from a finite sample, there is always a certain probability that the sample average is very different from the value being estimated (Bharat and Broder 1998). Our approach relies on our DMOZ corpus being a reliable sample of web pages, but it is a relatively small, finite subcorpus of half a million high-quality webpages from 2006. We have aimed to reduce the sampling error by repeating the estimate over a range of word frequencies, with 28 pivot words of which the frequencies are log-linearly spaced. We observe differences among the words (cf. Table 1; Fig. 3), but also see that their reported document counts follow the same overall trends (cf. Fig. 3).

### Future work

The unique perspective of our study is its longitude. Already in 1999, Rousseau remarked that collecting time series estimates should be an essential part of Internet research. The nine-year view visualized in Fig. 2 shows that our estimation is highly variable. It is likely that other estimation approaches, e.g. using link structure or result rankings, would show similar variance if they were carried out longitudinally. Future work should include comparing the different estimation methods over time periods, at least of a few years. The sustainability of this experiment is non-trivial and should be planned carefully, including a continuous monitoring of the proper functioning. The scripts that ran our experiment for nearly nine years, and are still running, had to be adapted to changes in the web interfaces of Google and Bing repeatedly. The time required for adapting the scripts after the detection of a change caused the loss of 6–7 % of all possible daily measurements.

Our study also opens up additional avenues for future research. For instance, we have tacitly assumed that a random selection of DMOZ pages represents “all languages”. With the proper language identification tools, by which we can identify a proper DMOZ subset of pages in a particular language, our method allows to focus on that language. This may well produce an estimate of the number of pages available on the Web in that language. Estimations for Dutch produce numbers close to two billion Web pages. Knowing how much data is available for a particular language, based on a seed corpus, is relevant background information for language engineering research and development that uses the web as a corpus (Kilgarriff and Grefenstette 2003).

Furthermore, we have not addressed the issue of overlap between search engines (Bharat and Broder 1998). If we could identify the overlap between Google and Bing at all points in time, we could generate an aggregate estimate of the sum of the two estimated index sizes, minus their overlap. In fact, we did measure the overlap between the two search engines at the beginning of our study in 2006 (de Kunder 2006). Based on querying the search engines with 784 log-lineary spaced pivot words and measuring the overlap in the returned results, 9.61 % of the URLs indexed by Google were not indexed by Microsoft Live Search, and 8.73 % vice versa; less than the 15 % lack of overlap reported by Spink et al. (2006). However, we did not update this overlap estimate and did not use it in the present study, as using the 2006 overlap between the two search engines would arguably not be suitable for continued use.

## Conclusions

We presented a novel method for estimating the daily number of webpages indexed by the Google and Bing web search engines. The method is based on comparing word frequencies from a known training corpus of web pages against hit counts reported by a search engine, and estimating the number of webpages indexed by the search engine through extrapolation. As we repeated the same procedure on a daily basis during nine years, we were able to observe that estimates of the numbers of pages indexed by the Google and Bing search engines both tend to vary dramatically over time; this variation is very different between the two. This result raises doubts about the reliability of previous one-off estimates of the size of the indexed Web. We find that much, if not all of this variability can be explained by changes in the indexing and ranking infrastructure of Google and Bing. This casts further doubt on whether Web search engines can be used reliably for cross-sectional webometric studies.

It has been pointed out before that “Googleology is bad science” (Kilgarriff 2007, p. 147), meaning that commercial search engines seem to exhibit variations in their functioning that do not naturally link to the corpus they claim to index (cf. our Fig. 2), and that there have been cases where the reported document counts were clearly inflated or otherwise false.^16^ Important future work lies in solving this unwanted lack of control over gathering data for scientific purpose.

## Footnotes

1 Formerly known as (Microsoft) Live Search until May 28, 2009, and as MSN Search until September 2006.

2 We base our estimates on words rather than on multi-word queries based on the findings of Uyar (2009).

3 DMOZ is also called the Open Directory Project, available at http://www.dmoz.org/, last visited December 1, 2015.

4 Available at https://catalog.ldc.upenn.edu/LDC2003T05, last visited December 1, 2015.

5 Available at http://trec.nist.gov/data/reuters/reuters.html, last visited December 1, 2015.

6 Downloaded on October 28, 2007.

7 Recent daily estimates produced by our method can be accessed through http://www.worldwidewebsize.com/. The time series data displayed in Fig. 2 are available online at http://toinebogers.com/?page_id=757.

8 Originally, we also sent the same 28 words to two other search engines that were discontinued at some point after 2006.

9 Available at http://googleblog.blogspot.com/2011/11/ten-algorithm-changes-on-inside-search.html, last visited December 1, 2015.

10 Available at http://googlewebmastercentral.blogspot.com/, last visited December 1, 2015.

11 Available at http://googleblog.blogspot.com/, last visited December 1, 2015.

12 Available at http://blogs.bing.com/, last visited December 1, 2015.

13 Available at http://searchenginewatch.com, last visited December 1, 2015.

14 A complete, numbered list of these events can be found at http://toinebogers.com/?page_id=757.

15 Available at http://www.bing.com/toolbox/bingsearchapi, last visited December 1, 2015.

16 For example, Kilgarriff (2007) points to the entries on “Yahoo’s missing pages” (2005) and “Crazy duplicates” (2006) in Jean Véronis’ blog at http://aixtal.blogspot.com.
